# Supplementary material for: Bidirectional control of fear memories by cerebellar neurons projecting to the ventrolateral periaqueductal grey
Source: Nat Commun. 2020 Oct 15;11:5207. doi: 10.1038/s41467-020-18953-0 (PMC7566591; doi:10.1038/s41467-020-18953-0)
Supplement: Supplementary file 1 — Supplementary Information [file 41467_2020_18953_MOESM1_ESM.pdf]

Supplementary Information

Bidirectional control of fear memories by cerebellar neurons projecting to the ventrolateral periaqueductal grey

Frontera et al., *Nature communications*

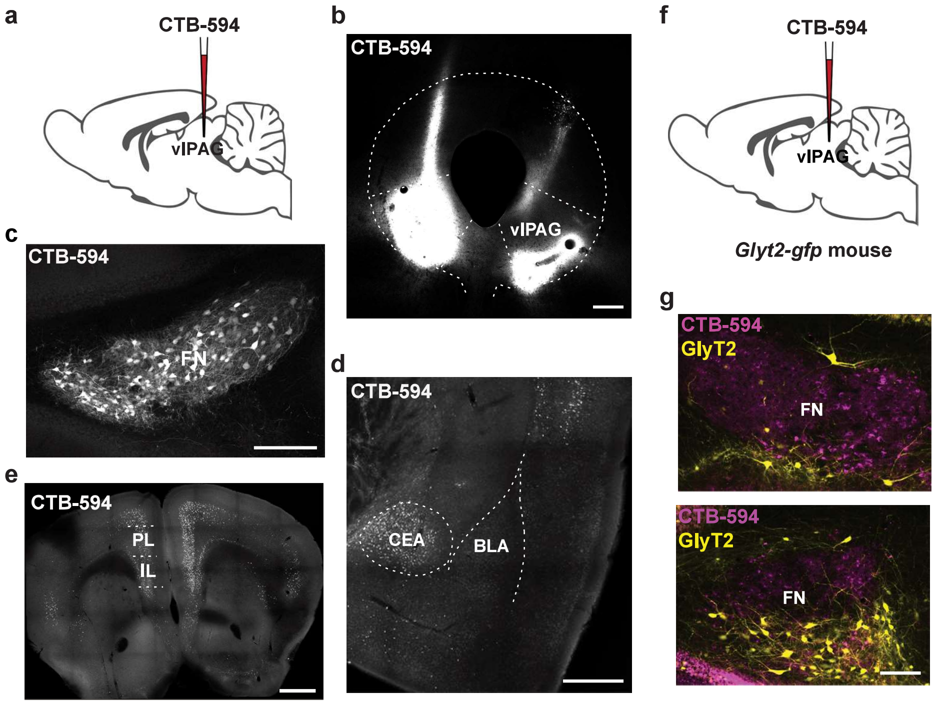

Supplementary Figure 1. vlPAG receives monosynaptic projections from FN, CEA, and mPFC. a, Retrograde tracing from vlPAG. **b,** Injection site of CTB-594 in the vlPAG (scale bar, 100 μm). **c-e,** vlPAG received monosynaptic projections from FN (scale bar, 200 μm), CEA (scale bar, 500 μm), and from the PL and IL areas of the mPFC (scale bar, 1mm). **f,** Retrograde injection of CTB-594 in vlPAG of glyt2-gfp mice line. **g,** Retrograde tracing from vlPAG into the FN did not co-localized with GlyT2+ cells (scale bar, 100 μm).

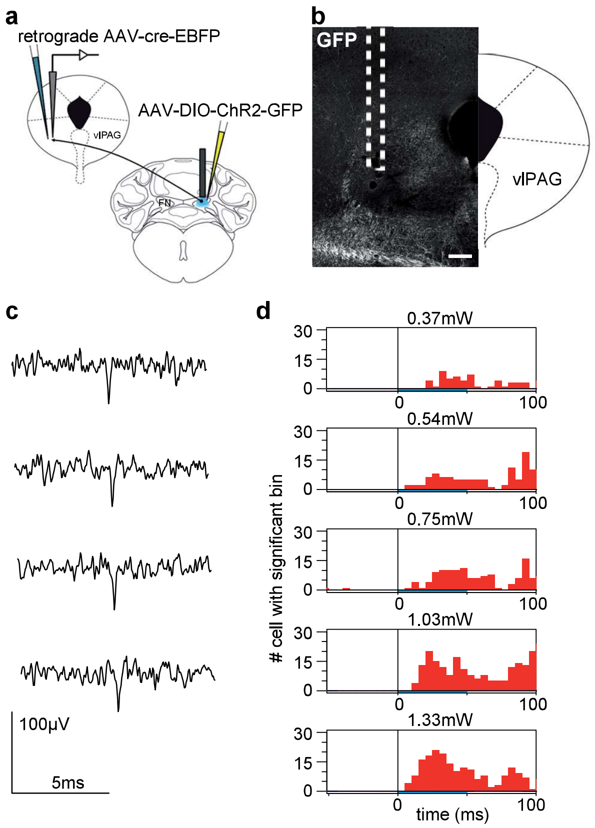

Supplementary Figure 2. Optogenetic stimulation of FN-vlPAG pathway and electrophysiological recordings in freely moving animals. a, Strategy to assess optogenetic stimulation in FN-vlPAG projecting neurons and electrophysiological recordings in the vlPAG. **b,** Example of electrode position in the vlPAG for the electrophysiological recordings (scale bar, 200 µm). **c,** same trace excerpts as fig 3b, after high-pass filtering. **d,** histogram of number of cells exhibiting a significant bin (z-score PSTH > 4) at each time around the stimulation (blue bar under the histogram) for the range of intensities used in fig 3a.

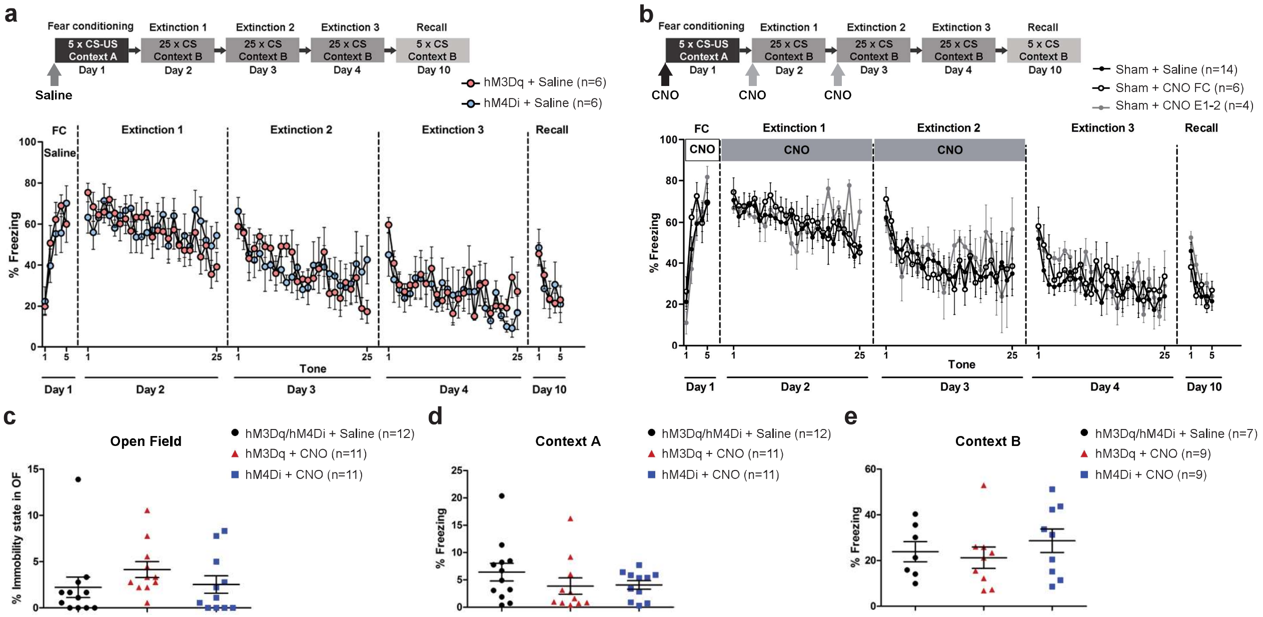

Supplementary Figure 3. DREADD expression or CNO administration do not affect freezing expression. a, No differences were found in fear conditioning and extinction between control mice injected with saline expressing hM3Dq and hM4Di (hM3Dq + Saline, n=6 mice; hM4Di + saline, n=6 mice) (FC: F(1,58)=0.40, P=0.529; Extinction 1: F(1,260)=0.28, P=0.599; Extinction 2: F(1,260)=0.01, P=0.938; Extinction 3: F(1,260)=1.94, P=0.165; Recall: F(1,54)=0.03, P=0.853; two-way ANOVA). Data are presented as mean values ± SEM. **b,** CNO had not effect per se on the freezing behavior in control sham mice (Sham + saline, n=14) either during fear conditioning (Sham + CNO FC, n=6 mice) (FC: F(1,95)=2.16, P=0.145; Extinction 1: F(1,434)=3.86, P=0.0501; Extinction 2: F(1,432)=0.18, P=0.674; Extinction 3: F(1,443)=3.58, P=0.059; Recall: F(1,85)=0.80, P=0.530; two-way ANOVA), or extinction sessions (Sham + CNO E1-2, n=4 mice) (FC: F(1,83)=0.10, P=0.757; Extinction 1: F(1,398)=2.74, P=0.099; Extinction 2: F(1,432)=0.18, P=0.674; Extinction 3: F(1,404)=3.36, P=0.067; Recall: F(1,85)=3.41, P=0.068; two-way ANOVA). Data are presented as mean values ± SEM. **c,** Chemogenetic activation or inhibition of FN-vlPAG pathway have not effect on immobility state in the open field (hM3Dq/ hM4Di + Saline, n=12 mice; hM3Dq + CNO, n=11 mice; hM4Di + CNO, n=11 mice; df=2, F=1.082, P=0.351, one-way ANOVA). Scatter dot plot with mean values ± SEM. **d,** Mice under chemogenetic inhibition or activation of FN-vlPAG pathway exhibited similar basal freezing levels in Context A before fear conditioning (hM3Dq/ hM4Di + Saline, n=12; hM3Dq + CNO, n=11; hM4Di + CNO, n=11; df=2, F=1.108, P=0.343, one-way ANOVA). Scatter dot plot with mean values ± SEM. **e,** Mice under chemogenetic inhibition or activation of FN-vlPAG pathway during extinction exhibited similar basal freezing levels to the Context B before extinction training (hM3Dq/ hM4Di + Saline, n=7; hM3Dq + CNO, n=9; hM4Di + CNO, n=9; df=2, F=0.652, P=0.531, one-way ANOVA). Scatter dot plot with mean values ± SEM. Source data are provided as Source Data file.

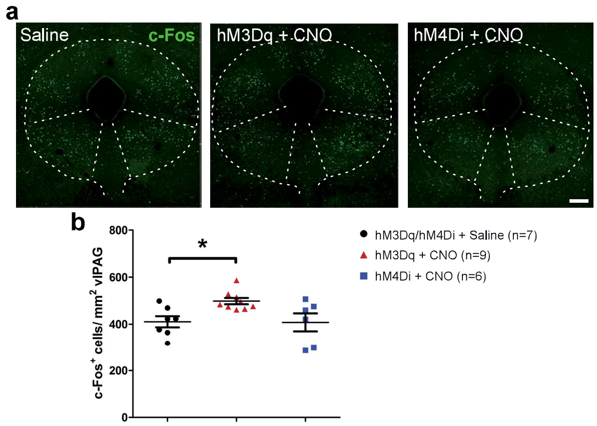

Supplementary Figure 4. Stimulation of glutamatergic FN-vlPAG projections during fear conditioning increase neuronal activity in the vlPAG. a, C-Fos expression in vlPAG after fear conditioning under bilateral FN-vlPAG chemogenetic stimulation and inhibition (scale bar, 200 µm). **b,** C-Fos expression increased in vlPAG under stimulation of FN-vlPAG pathway during fear conditioning compared to the control group (hM3Dq/ hM4Di + Saline, n=7 mice; hM3Dq + CNO, n=9 mice; hM4Di + CNO, n=6 mice) (df=2, F=5.003, P=0.018, two-tailed, Newman-Keuls Multiple comparison test). Scatter dot plot with mean values ± SEM, *P<0.05. Source data are provided as Source Data file.

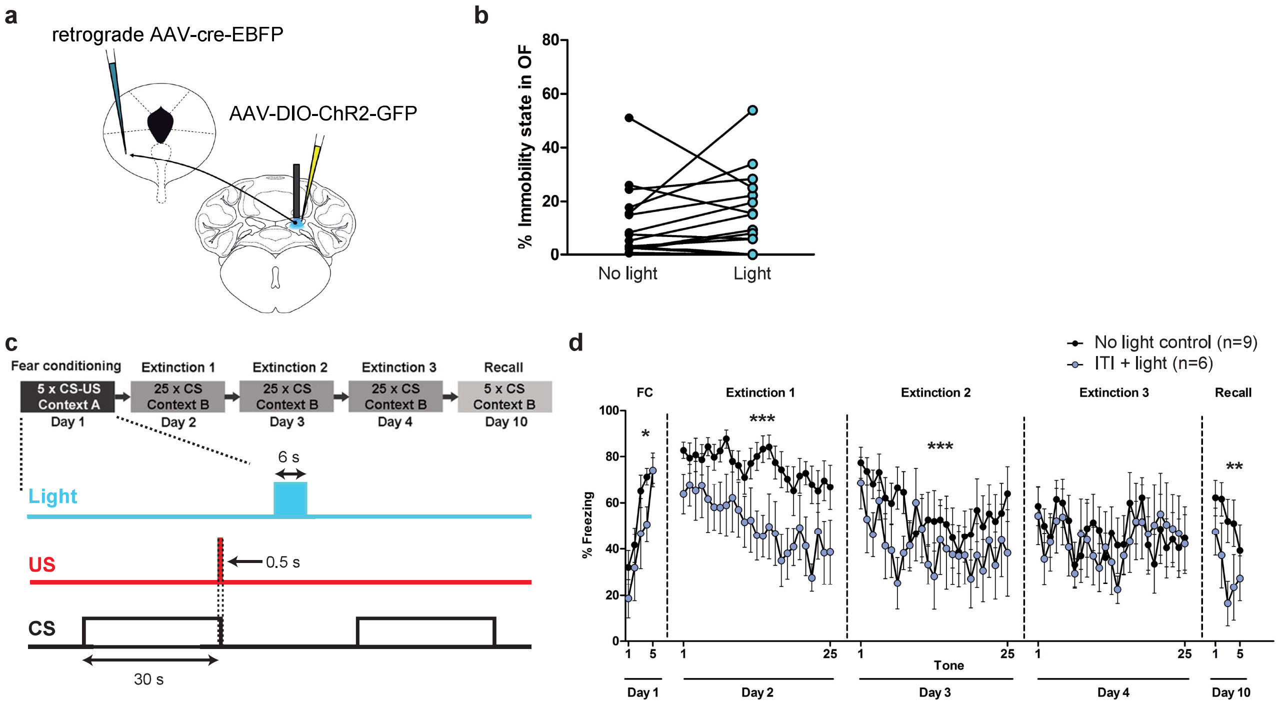

Supplementary Figure 5. FN-vlPAG pathway modulates fear learning. a, Optogenetic stimulation of FN-vlPAG projections expressing cre-dependent ChR2-GFP strategy. **b,** Light stimulation of FN-vlPAG projecting neurons have not effect on immobility state in the open field. Mice expressing ChR2 in FN-vlPAG projecting neurons were exposed to an open field 5 min without light and 5 min with light stimulation (n=15, P=0.299, two-tailed, paired t-test). **c,** Optogenetic stimulation protocol of FN-vlPAG projecting neurons randomly within the ITI during fear conditioning. **d,** Freezing levels during fear conditioning and extinction sessions in control (no light control, n=9) and FN-vlPAG stimulated mice (ITI + light, n=6). Mice that received light stimulation during ITI exhibited a lower curve of learning during fear conditioning and lower fear response during retrieval of fear memory and extinction sessions 1 and 2, compared to the mice that were not stimulated (FC: F(1,63)=4.99, P= 0.0291; Extinction 1: F(1,330)=102.01, P<0.0001; Extinction 2: F(1,305)=18.45, P<0.0001; Recall: F(1,65)=9.88, P=0.025, two-way ANOVA). *P<0.05, ** P<0.01, *** P<0.001. Data are presented as mean values ± SEM. Source data are provided as Source Data file.

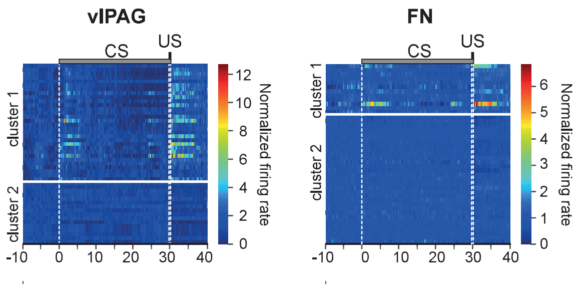

Supplementary Figure 6. Average of firing during conditioning for cluster 1 and cluster 2 units. Time axis is relative to CS onset; each line corresponds to the average of the 5 CS-US trials for a single FN or vlPAG unit; bin=200ms. The PSTHs are normalized by dividing by the baseline firing rate. The ordering of the cells is the same as in Fig. 5 f. Source data are provided as Source Data file.

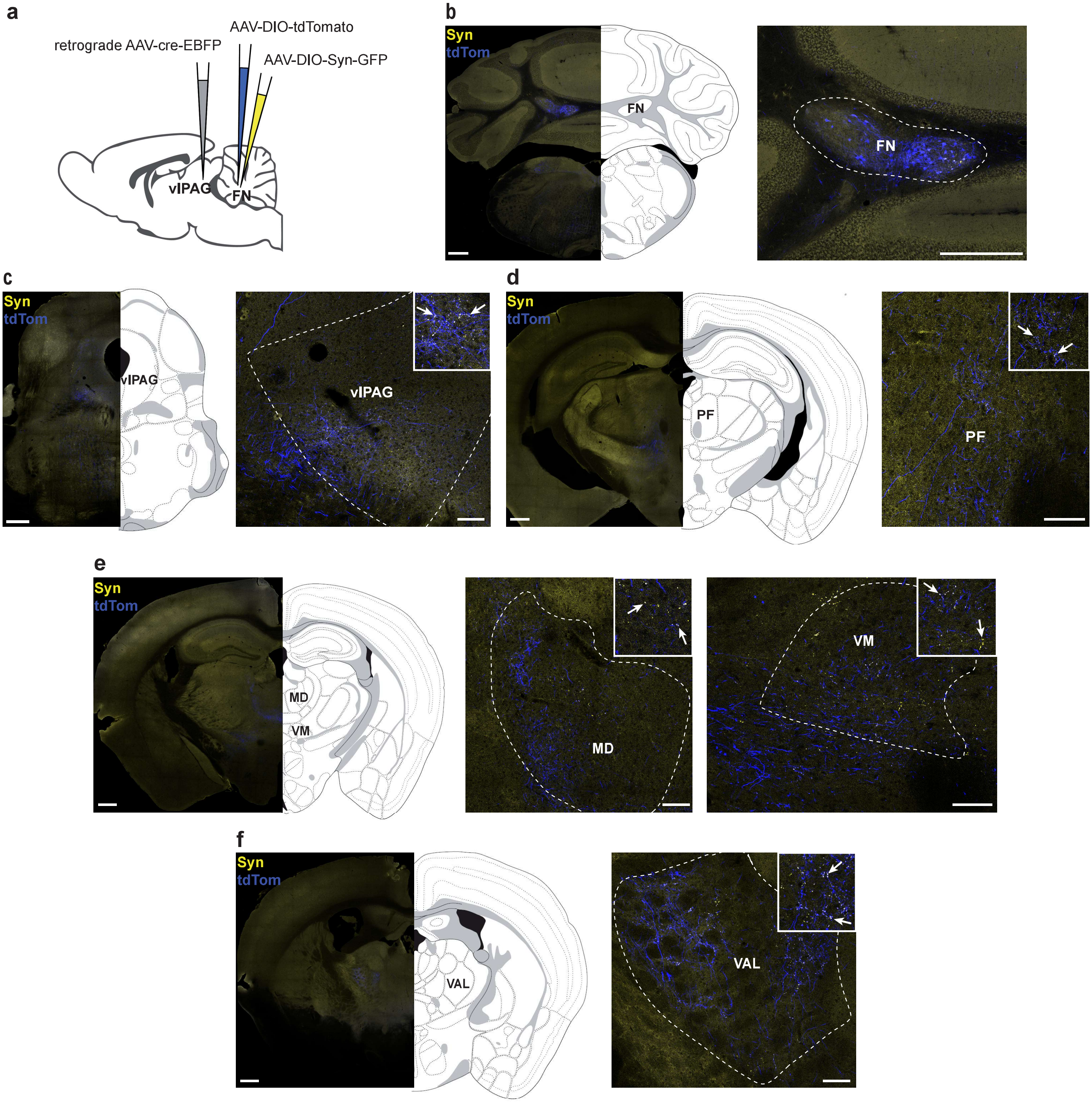

Supplementary Figure 7. FN-vlPAG axon collaterals to different nuclei in the thalamus. a, Tracing of axon collaterals from FN-vlPAG projecting neurons by expression of retrograde AAV-cre-EBFP in vlPAG, AAV-DIO-Syn-GFP and AAV-DIO-tdTomato in FN. **b,** FN neurons projecting to vlPAG expressing cre-dependent Synaptophysin-GFP and tdTomato (scale bar, 500 µm). **c,** Midbrain section (left panel, scale bar 500 µm) exhibiting synaptic boutons in FN inputs to vlPAG (right panel, scale bar 100 µm), inset shows zoom-in on synaptic boutons (arrows). **d,** Posterior thalamus section (left panel, scale bar 500 µm) showing axon collaterals with synaptic boutons n PF (right panel, scale bar 100 µm). Inset shows zoom-in on synaptic boutons (arrows). **e,** Medial thalamus section (left panel, scale bar 500 µm) showing axon collaterals with synaptic boutons in MD (center panel, scale bar 100 µm) and VM (right panel, scale bar 100 µm). Insets show zoom-in on synaptic boutons (arrows). **f,** Anterior thalamus section (left panel, scale bar 500 µm) showing axon collaterals with synaptic boutons in VAL (right panel, scale bar 100 µm). Inset shows zoom-in on synaptic boutons (arrows).

a

Open field				
Group	Total distance moved (cm)	Frequency of entries to centre	Time in centre (%)	Distance moved in centre (cm)
hM3q/hM4Di + saline	5941 ± 302	130 ± 7	29.7 ± 2.9	1916 ± 164
hM3Dq + CNO	5717 ± 238	108 ± 6	23.4 ± 1.7	1529 ± 116
hM4Di + CNO	6028 ± 277	117 ± 6	25.8 ± 1.7	1714 ± 124
	One way anova, p=0.673	One way anova, p=0.085	One way anova, p=0.116	One way anova, p=0.141

Elevated plus maze				
	Total distance moved (cm)	Frequency in open arms	Time in open arms (%)	Frequency in closed arms
hM3q/hM4Di + saline	1426 ± 57	6.58 ± 0.87	6.51 ± 1.48	22.0 ± 4.4
hM3Dq + CNO	1599 ± 93	10.93 ± 1.69	7.83 ± 1.81	19.7 ± 1.7
hM4Di + CNO	1659 ± 71	9.21 ± 1.45	7.95 ± 1.41	27.5 ± 4.7
	One way anova, p=0.082	One way anova, p=0.119	One way anova, p=0.774	One way anova, p=0.403

Dark light box			
	Frequency in light zone	Time in light zone (%)	Latency to enter in dark zone (s)
hM3q/hM4Di + saline	8.50 ± 1.26	28.0 ± 3.4	16.0 ± 3.0
hM3Dq + CNO	11.3 ± 0.59	30.4 ± 3.7	14.8 ± 4.0
hM4Di + CNO	8.83 ± 0.94	27.1 ± 7.2	18.4 ± 4.2
	One way anova, p=0.075	One way anova, p=0.058	One way anova, p=0.809

b

Open field				
Group	Total distance moved (cm)	Frequency of entries to centre	Time in centre (%)	Distance moved in centre (cm)
hM3q/hM4Di + saline	5610 ± 357	76 ± 5	14.3 ± 1.8	1268 ± 163
hM3Dq + CNO	5634 ± 248	90 ± 10	16.8 ± 1.5	1333 ± 278
hM4Di + CNO	6040 ± 440	90 ± 16	19.8 ± 6.2	1164 ± 126
	One way anova, p=0.706	One way anova, p=0.402	One way anova, p=0.363	One way anova, p=0.821

Elevated plus maze				
	Total distance moved (cm)	Frequency in open arms	Time in open arms (%)	Frequency in closed arms
hM3q/hM4Di + saline	1723 ± 131	8.17 ± 1.33	9.89 ± 2.44	23.6 ± 1.3
hM3Dq + CNO	1292 ± 75	4.67 ± 0.33 *	5.88 ± 1.02	14.0 ± 2.5**
hM4Di + CNO	1807 ± 103	13.75 ± 0.63	14.0 ± 2.48	22.8 ± 1.2
	One way anova, p=0.096	One way anova, p=0.019	One way anova, p=0.268	One way anova, p=0.005

Dark light box			
	Frequency in light zone	Time in light zone (%)	Latency to enter in dark zone (s)
hM3q/hM4Di + saline	23.5 ± 2.8	51.0 ± 6.1	10.8 ± 1.8
hM3Dq + CNO	9.0 ± 1.5**	40.8 ± 17.9	8.0 ± 1.5
hM4Di + CNO	24.8 ± 1.4	41.8 ± 3.0	8.7 ± 1.0
	One way anova, p=0.005	One way anova, p=0.466	One way anova, p=0.513

c

Open field				
Group	Total distance moved (cm)	Frequency of entries to centre	Time in centre (%)	Distance moved in centre (cm)
SHAM + saline	5853 ± 391	115 ± 173	21.7 ± 5.4	1450 ± 326
SHAM + CNO	5301 ± 313	110 ± 10	21.7 ± 2.9	1389 ± 185
	t test, p=0.289	t test, p=0.795	t test, p=0.995	t test, p=0.863

Elevated plus maze				
	Total distance moved (cm)	Frequency in open arms	Time in open arms (%)	Frequency in closed arms
SHAM + saline	1570 ± 142	7.17 ± 0.79	8.32 ± 2.04	20.0 ± 2.4
SHAM + CNO	1438 ± 60	7.89 ± 1.18	6.98 ± 1.45	17.9 ± 1.6
	t test, p=0.351	t test, p=0.659	t test, p=0.591	t test, p=0.457

Dark light box			
	Frequency in light zone	Time in light zone (%)	Latency to enter in dark zone (s)
SHAM + saline	10.0 ± 0.97	22.3 ± 4.5	13.2 ± 4.3
SHAM + CNO	11.1 ± 1.3	27.2 ± 4.3	21.2 ± 7.8
	t test, p=0.559	t test, p=0.461	t test, p=0.450

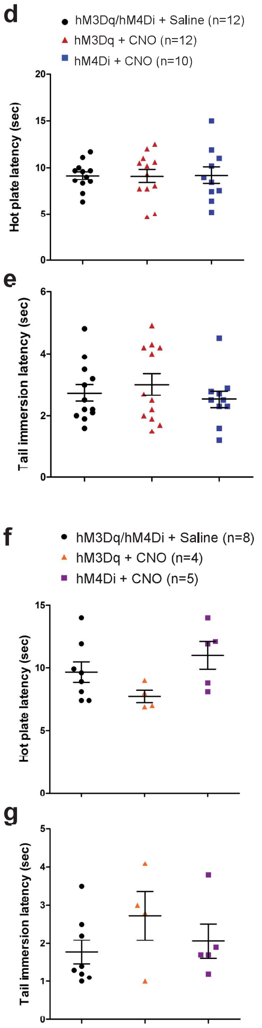

Supplementary Figure 8. FN-PF, but not FN-vlPAG, is involved in anxiety like behavior, and both have not effect on pain sensitivity. a, FN-vlPAG chemogenetic activation or inhibition did not induce anxiety-like behavior (P>0.05, one-way ANOVA). Data are presented as mean values ± SEM. **b,** FN-PF activation induced an increase in anxiogenic like behavior in the Elevated plus maze and in the Dark light box tests (*P<0.05, **P<0.01, Bonferroni post-hoc test). Data are presented as mean values ± SEM. **c,** CNO administration had not effect on anxiety-like behavior in absence of DREADD receptors in sham mice (P>0.05, two-tailed, t test), data are presented as mean values ± SEM. **d-e,** FN-vlPAG stimulation (hM3Dq + CNO, n=12) or inhibition (hM4Di + CNO, n=10) had not significant effect on hot plate (df=2, F=0.0039, P=0.996, one-way ANOVA) and tail immersion (df=2, F=0.591, P=0.559, one-way ANOVA) tests compared to the control group (hM3Dq/ hM4Di + Saline, n=12). Data are presented as scatter dot plot with mean values ± SEM. **f-g,** FN-PF stimulation (hM3Dq + CNO, n=4) or inhibition (hM4Di + CNO, n=5) had not significant effect on hot plate (df=2, F=2.57,P=0.112, one-way ANOVA) and tail immersion (df=2, F=1.169, P=0.339, one-way ANOVA) tests, compared to the control group (hM3Dq/ hM4Di + Saline, n=8). Data are presented as scatter dot plot with mean values ± SEM. Source data are provided as Source Data file.

Wilcoxon Test on Transfer Entropy Polarity Gq + SAL

Transfer Entropy 1	Mean	SD	Transfer Entropy 2	Mean	SD	Wilcoxon	p	Significance
FN firing→ vlPAG firing	0.571	0.161	vlPAG firing → FN firing	0.419	0.163	10.002	< 0.001	***
FN firing → Motor activity	0.600	0.186	Motor activity → FN firing	0.484	0.130	3.517	0.002	**
Motor activity → vlPAG firing	0.520	0.165	vlPAG firing → Motor activity	0.324	0.172	450.000	< 0.001	***

Wilcoxon Test on Transfer Entropy Polarity Gq + CNO

Transfer Entropy 1	Mean	SD	Transfer Entropy 2	Mean	SD	Wilcoxon	p	Significance
FN firing→ vlPAG firing	0.665	0.174	vlPAG firing → FN firing	0.253	0.096	443.000	< 0.001	***
FN firing → Motor activity	0.653	0.173	Motor activity → FN firing	0.503	0.137	624.000	< 0.001	***
Motor activity → vlPAG firing	0.528	0.129	vlPAG firing → Motor activity	0.242	0.096	16.190	< 0.001	***

ANOVA FNunique Cluster 1(White correction for heteroscedasticity)

Factor	df	F	p	Significance
Stage	2	116.08	< 0.001	***
Treatment	1	2.515	0.115	n.s.
Stage:Treatment	2	79.085	< 0.001	***

Tukey Posthoc Test Gq + SAL

Stage 1	Stage 2	Mean Difference	CI 2.5 %	CI 97.5 %	p	Significance
Baseline	Conditioning	18.304	14.6001	22.000	< 0.001	***
Baseline	After	9.535	5.836	13.235	< 0.001	***
Conditioning	After	-8.764	-12.464	-5.065	< 0.001	***

Tukey Posthoc Test Gq + CNO

Stage 1	Stage 2	Mean Difference	CI 2.5 %	CI 97.5 %	p	Significance
Baseline	Conditioning	2.962	1.030	4.896	0.001	**
Baseline	After	0.477	-1.456	2.410	0.809	n.s.
Conditioning	After	-2.486	-4.419	-0.553	0.008	**

Mann Whitney Test FNunique Cluster 1 Basline

Group 1	Group 2	Statstic	p	Significance
Gq + SAL	Gq + CNO	13.000	< 0.001	***

ANOVA MotorActivityunique Cluster 1(White correction for heteroscedasticity`

Factor	df	F	p	Significance
Stage	2	260.426	< 0.001	***
Treatment	1	29.167	< 0.001	***
Stage:Treatment	2	149.616	< 0.001	***

Tukey Posthoc Test Gq + SAL

Stage 1	Stage 2	Mean Difference	CI 2.5 %	CI 97.5 %	p	Significance
Baseline	Conditioning	-47.260	-54.052	-40.468	< 0.001	***
Baseline	After	-32.026	-38.818	-25.234	< 0.001	***
Conditioning	After	15.234	8.441	22.026	< 0.001	***

Tukey Posthoc Test Gq + CNO

Stage 1	Stage 2	Mean Difference	CI 2.5 %	CI 97.5 %	p	Significance
Baseline	Conditioning	-10.088	-14.0923	-6.084	<0.001	***
Baseline	After	-3.273	-7.277	0.731	0.132	n.s.
Conditioning	After	6.815	2.811	10.819	<0.001	***

Mann Whitney Test MotorActivityunique Cluster 1 Baseline

Group 1	Group 2	Statstic	p	Significance
Gq + SAL	Gq + CNO	782.000	< 0.001	***

ANOVA FNunique Cluster 1(White correction for heteroscedasticity)

Factor	df	F	p	Significance
Stage	2	6.646	0.001	**
Treatment	1	25.758395	< 0.001	***
Stage:Treatment	2	1.768	0.173	n.s.

Tukey Posthoc Test Gq + SAL

Stage 1	Stage 2	Mean Difference	CI 2.5 %	CI 97.5 %	p	Significance
Baseline	Conditioning	3.226	-0.133	6.585	0.062	n.s.
Baseline	After	4.326	0.967	7.685	0.009	**
Conditioning	After	1.010	-2.259	4.459	0.694	n.s.

Tukey Posthoc Test Gq + CNO

Stage 1	Stage 2	Mean Difference	CI 2.5 %	CI 97.5 %	p	Significance
Baseline	Conditioning	-2.558	-6.932	1.816	0.353	n.s.
Baseline	After	3.441	-0.933	7.815	0.157	n.s.
Conditioning	After	5.999	1.625	10.373	0.004	**

Mann Whitney Test FNunique Cluster 2 Baseline

Group 1	Group 2	Statstic	p	Significance
Gq + SAL	Gq + CNO	108.000	< 0.001	***

ANOVA MotorActivityunique Cluster 1(White correction for heteroscedastic

Factor	df	F	p	Significance
Stage	2	260.426	0.478	n.s.
Treatment	1	29.167	< 0.001	***
Stage:Treatment	2	149.616	0.041	*

Tukey Posthoc Test Gq + SAL

Stage 1	Stage 2	Mean Difference	CI 2.5 %	CI 97.5 %	p	Significance
Baseline	Conditioning	-9.044	-18.909	0.821	0.078	n.s.
Baseline	After	-11.187	-21.052	-1.322	0.023	*
Conditioning	After	-2.143	-12.008	7.722	0.846	n.s.

Tukey Posthoc Test Gq + CNO

Stage 1	Stage 2	Mean Difference	CI 2.5 %	CI 97.5 %	p	Significance
Baseline	Conditioning	5.801	-3.408	15.011	0.299	n.s.
Baseline	After	2.347	-6.862	11.557	0.800	n.s.
Conditioning	After	-3.454	-12.664	5.755	0.637	n.s.

Mann Whitney Test MotorActivityunique Cluster 2 Baseline

Group 1	Group 2	Statstic	p	Significance
Gq + SAL	Gq + CNO	685.000	< 0.001	***

Supplementary Table 1. Statistics and P values for each statistical comparison in Figure 5 j, l-m. Wilcoxon tests, Mann-Whitney tests and Tukey Posthoc tests are two-sided.
