# Supplementary material for: Bidirectional control of fear memories by cerebellar neurons projecting to the ventrolateral periaqueductal grey
Source: Nat Commun. 2020 Oct 15;11:5207. doi: 10.1038/s41467-020-18953-0 (PMC7566591; doi:10.1038/s41467-020-18953-0)
Supplement: Supplementary file 3 — Reporting Summary [file 41467_2020_18953_MOESM3_ESM.pdf]

nature research

Corresponding author(s): Daniela Popa

Last updated by author(s): August 24, 2020

Reporting Summary

Nature Research wishes to improve the reproducibility of the work that we publish. This form provides structure for consistency and transparency in reporting. For further information on Nature Research policies, see our Editorial Policies and the Editorial Policy Checklist.

Statistics

For all statistical analyses, confirm that the following items are present in the figure legend, table legend, main text, or Methods section.

n/a	Confirmed	
☐	☒	The exact sample size (*n*) for each experimental group/condition, given as a discrete number and unit of measurement
☐	☒	A statement on whether measurements were taken from distinct samples or whether the same sample was measured repeatedly
☐	☒	The statistical test(s) used AND whether they are one- or two-sided<br>*Only common tests should be described solely by name; describe more complex techniques in the Methods section.*
☒	☐	A description of all covariates tested
☐	☒	A description of any assumptions or corrections, such as tests of normality and adjustment for multiple comparisons
☐	☒	A full description of the statistical parameters including central tendency (e.g. means) or other basic estimates (e.g. regression coefficient) AND variation (e.g. standard deviation) or associated estimates of uncertainty (e.g. confidence intervals)
☐	☒	For null hypothesis testing, the test statistic (e.g. *F*, *t*, *r*) with confidence intervals, effect sizes, degrees of freedom and *P* value noted<br>*Give P values as exact values whenever suitable.*
☒	☐	For Bayesian analysis, information on the choice of priors and Markov chain Monte Carlo settings
☒	☐	For hierarchical and complex designs, identification of the appropriate level for tests and full reporting of outcomes
☒	☐	Estimates of effect sizes (e.g. Cohen's *d*, Pearson's *r*), indicating how they were calculated

Our web collection on statistics for biologists contains articles on many of the points above.

Software and code

Policy information about availability of computer code

Data collection	Synapse Suite version 95 (Tucker-Davis Technologies), Multi Channel Experimenter version 2.15 (Multi Channel Systems)
Data analysis	Matlab R2006a, Prism Graphpad 5, R 3.6.3, RStudio 1.2.5, Python3, ImageJ-win64, Ethovision XT 14, PyiF v0.1, idtxl v1.0

For manuscripts utilizing custom algorithms or software that are central to the research but not yet described in published literature, software must be made available to editors and reviewers. We strongly encourage code deposition in a community repository (e.g. GitHub). See the Nature Research guidelines for submitting code & software for further information.

Data

Policy information about availability of data

All manuscripts must include a data availability statement. This statement should provide the following information, where applicable:

- Accession codes, unique identifiers, or web links for publicly available datasets
- A list of figures that have associated raw data
- A description of any restrictions on data availability

The data that support the findings of this study (Fig. 1-9, Supplementary Figs.1-8) is available from the corresponding author upon reasonable request.
For additional information please refer to the corresponding Life Sciences Reporting Summary.

Field-specific reporting

April 2020

Life sciences study design

All studies must disclose on these points even when the disclosure is negative.

Sample size	Our sample-size are consistent with sample size's in the fear conditioning literature (e.g. Karalis et al., 2016, Nat Neurosci. doi: 10.1038/nn.4251; Grewe et al., 2017, Nature. doi: 10.1038/nature21682; Grössl et al., 2019, Nat Neurosci. doi:10.1038/s41593-018-0174-5). Sample size was determined to be adequate based on the magnitude and consistency of measurable differences between groups. The sample size (n) of each experiment is provided in the corresponding figure captions in the main manuscript and in materials and methods section. Sample sizes were chosen to support meaningful conclusions in accordance with ethical committee requirements to limit as much as possible the use of animals.
Data exclusions	Few animals were excluded from electrophysiological and optogenetic analysis when the optic fiber or the electrode was outside of the target area after histological verifications.
Replication	Each experiment presented in the paper was repeated in multiple animals in at least 3 series, each series containing animals assigned to different groups. The replication of each experiment was succesful, and each group thus derives from multiple independent experiments.
Randomization	Animals were assigned randomly to experimental and control groups, and within animal controls were performed wherever possible.
Blinding	For all the experiments in this study, animals were assigned randomly to each experimental group and the investigators were not blinded to group allocation during the data collection because it was not possible due to the investigators had to do the administration of the different treatment/drug during the experiment. Blinding was used during the analysis of freezing behavior for fear conditioning and extinction experiments as well as for all the other behavioral tests (anxiety, hot plate and tail immersion tests). Blinding was also used for the analyses of the other experiments, such as for the electrophysiological recordings and quantification of histology.

Reporting for specific materials, systems and methods

We require information from authors about some types of materials, experimental systems and methods used in many studies. Here, indicate whether each material, system or method listed is relevant to your study. If you are not sure if a list item applies to your research, read the appropriate section before selecting a response.

Materials & experimental systems

n/a	Involved in the study	
☐	☒	Antibodies
☒	☐	Eukaryotic cell lines
☒	☐	Palaeontology and archaeology
☐	☒	Animals and other organisms
☒	☐	Human research participants
☒	☐	Clinical data
☒	☐	Dual use research of concern

Methods

n/a	Involved in the study	
☒	☐	ChIP-seq
☒	☐	Flow cytometry
☒	☐	MRI-based neuroimaging

Antibodies

Antibodies used	This work used donkey anti-mouse IgG conjugated to Alexa Fluor 488 (Invitrogen #A-21202) or to Alexa 555 (Invitrogen #A-31570), goat anti-rabbit IgG- FITC (Jackson Immunoresearch AB_2337972, #111-095-003), mouse anti-GAD67 (Milipore #MAB5406, clone 1G10.2), rabbit polyclonal anti-c-fos (Milipore #ABE457, clone 2G9C3).
Validation	Mouse anti-GAD67 (Milipore #MAB5406, clone 1G10.2) validated for use in IH, IH(P), WB for the detection of GAD67. Specific reaction with the 67kDa isoform of Glutamate Decarboxylase (GAD67) of rat, mouse and human origins (e.g. Bassant et al., 2005. PMID: 15728843 DOI: 10.1523/JNEUROSCI.4619-04.2005).<br>Rabbit polyclonal anti-c-fos (Milipore #ABE457, clone 2G9C3) validated in WB & IHC. Demonstrated to react with Human and Rat. Predicted to react with Mouse, Rhesus Macaque, Bovine, Sheep, Porcine, and Chimpanzee based on 100% sequence homology. (Kim et al., 2014. PMID: 24880214 DOI: 10.1038/nn.3725).

Animals and other organisms

Policy information about studies involving animals; ARRIVE guidelines recommended for reporting animal research

Laboratory animals	Details on the mice used in this study have been included in the Methods section of this manuscript. C57BL6/N male mice, 8 to 12 week-old, wild-type from Charles River Laboratories or mutant male mice Vglut2-cre and Glyt2-GFP from an in-house colony (IBENS, Paris, France) were used in this study.
Wild animals	No wild animals were used in the study.
Field-collected samples	No field collected samples were used in the study.

Ethics oversight	All animal procedures were performed in accordance with the recommendations contained in the European Community Council Directives (authorization number APAFIS#1334-2015070818367911 v3).

Note that full information on the approval of the study protocol must also be provided in the manuscript.
